# Association between obese phenotypes and risk of carotid artery plaque among chinese male railway drivers

**DOI:** 10.1186/s12889-022-14253-y

**Published:** 2022-10-05

**Authors:** Jia Pan, Zihang Wang, Chaohui Dong, Bo Yang, Lei Tang, Peng Jia, Shujuan Yang, Honglian Zeng

**Affiliations:** 1 Department of Health Management Center, Affiliated Hospital of Chengdu University , Chengdu, Sichuan China; 2 West China School of Public Health and West China Fourth Hospital, Sichuan University, Chengdu, Sichuan China; 3 School of Resource and Environmental Sciences, Wuhan University, Wuhan, China; 4 International Institute of Spatial Lifecourse Health (ISLE), Wuhan University, Wuhan, China

**Keywords:** Metabolic abnormality, Obese phenotypes, Carotid artery plaque, Male railway drivers

## Abstract

**Background:**

China has the world’s highest rail transportation network density, and the prevalence of obesity among railway workers in China is more than twice that of adults in the world. Carotid artery plaque (CAP) is a simple and noninvasive predictor of early atherosclerosis, while the association between different obese phenotypes and CAP risk among Chinese male railway drivers is unclear.

**Methods:**

This cross-sectional study was performed among 8,645 Chinese male railway drivers. Obese phenotypes were assessed based on the obesity status (the body mass index ≥ 28 kg/m^2^ as obesity vs. < 28 kg/m^2^ as non-obesity) and metabolic status (metabolically healthy vs. metabolically unhealthy). Metabolically unhealthy was defined as the presence of at least one dysfunction, including elevated blood pressure, elevated fasting blood glucose, elevated triglyceride, and reduced high-density-lipoprotein cholesterol. Four obese phenotypes were defined based on the body mass index and metabolic status, i.e., metabolically healthy non-obesity (MHNO), metabolically healthy obesity (MHO), metabolically unhealthy obesity (MUO), and metabolically unhealthy non-obesity (MUNO). Multivariable logistic regression was employed to estimate the association between different obese phenotypes and the risk of CAP. Subgroup analysis was performed to examine the variation of the association by age, circadian rhythm disorders, and history of smoking and drinking.

**Results:**

The prevalence of CAP among male railway drivers in MHO, MUO, MUNO, and MHNO was 8.75%, 18.67%, 17.82%, and 5.36%, respectively. Compared to those with MHNO, an increased risk for CAP was observed among those with MHO (OR = 2.18, 95% CI: 0.82, 5.10), MUO (OR = 1.78, 95% CI:1.44, 2.21), and MUNO (OR = 2.20, 95% CI: 1.67, 2.89). The subgroup analysis showed that both of the metabolically unhealthy groups (MUNO and MUO) aged < 45 years were prone to a higher risk of CAP (for the MUNO group, OR = 4.27, 95% CI:2.71, 7.10; for the MUO group, OR = 4.00, 95%CI: 2.26, 7.17).

**Conclusion:**

The obese phenotypes are associated with CAP risk in male railway drivers, especially those with metabolically unhealthy conditions aged < 45 years.

## Introduction

China has the world’s highest rail transportation network density [1]. The increasing traffic volume results in a heavy workload for railway drivers in China. Compared to workers in other occupations, railway drivers have longer working hours, less physical activity, extreme mental stress, poor sleep quality, and circadian rhythm disorders [1]. These occupational characteristics lead to a higher incidence of obesity [2, 3]. The prevalence of obesity among railway workers in China is more than twice that of adults globally [4, 5].

Obesity is a growing global public health issue [5], and one of the leading causes of cardiovascular disease (CVD) worldwide [6]. There is an increasing concern about the health and well-being of railway workers, as their safety is of utmost importance in maintaining the country’s transportation infrastructure. Therefore, reducing the number of obese workers in critical positions, such as train operators, is an urgent priority for the transportation industry. Obesity is considered a pivotal contributor to metabolic abnormalities [7]. It is associated with a constellation of metabolic abnormalities, including glucose abnormalities, high blood pressure, and high triglycerides, all of which are considered risk factors for CVD [8–10]. However, only 10-30% of obese individuals are reported to be metabolically healthy [11]. According to National Cholesterol Education Program Adult Treatment Panel III (NCEP ATP III) criteria [12, 13], four types of metabolic obese phenotypes have been well described, including metabolically healthy obesity (MHO), metabolically unhealthy obesity (MUO), metabolically healthy non-obesity (MHNO), and metabolically unhealthy non-obesity (MUNO). These metabolic obese phenotypes may be deemed more accurate predictors of CVD risk than obesity alone [14].

Carotid artery plaque (CAP), as detected by carotid ultrasound, is considered a simple and noninvasive predictor of early atherosclerosis and CVD [15, 16]. A cohort study indicated that CAP was associated with incident CVD events after adjustment for traditional CVD risk factors [17]. Another study reported an association of different obese phenotypes with CAP events in a Chinese population [18]. Nevertheless, few studies reported the association between metabolic obese phenotypes and the risk of CAP in railway drivers with a high prevalence of unhealthy lifestyle behaviors.

In this cross-sectional study based on a large sample of male railway drivers in southwest China, we aim to investigate the association between obese phenotypes and CAP risk, as well as the modification effect of age, circadian rhythms, and history of drinking and smoking on their associations. The findings could contribute to a better understanding of the role of obese phenotypes in the development of CVD among male railway drivers, and identify the groups of participants that may be at high risk for CAP.

## Methods

### Study design and participants

This was a cross-sectional study recruiting 14,354 male railway drivers from the Chengdu Bureau of China Railway Administration, including 50 railway stations in Sichuan Province, Guizhou Province, and Chongqing. All the male railway workers ≥ 18 years old underwent physical examination in the Affiliated Hospital of Chengdu University between January and December 2019.

### Inclusion and exclusion criteria

The on-job railway drivers who received physical examinations were included in this study. Exclusion criteria were participants with incomplete clinical information (e.g., blood pressure, fasting blood glucose, blood lipid, body mass index, etc.) and a history of severe diseases (e.g., renal or liver failure, and malignant). Finally, 8,645 male railway drivers were included in this survey.

All individuals voluntarily participated in this study, and their informed consent was obtained before the survey.

### Data collection and measurement

#### Definitions of metabolic status and body weight

According to the NCEP ATP III criteria, metabolically unhealthy parameters were defined as follows [12]: (1) elevated blood pressure: systolic blood pressure (SBP) ≥ 130 mmHg or diastolic blood pressure (DBP) ≥ 85 mmHg or using antihypertensive medications; (2) elevated fasting blood glucose (FBG): FBG level ≥ 5.60 mmol/L or on antidiabetic treatment; (3) elevated triglyceride (TG): TG level ≥ 1.7 mmol/L or using lipid-lowering medications; and (4) reduced high-density lipoprotein cholesterol(HDL-C): HDL-C level < 1.04 mmol/L, or using lipid-lowering medications. Obesity was defined as a body mass index (BMI) ≥ 28 kg/m^2^ based on the criteria developed by the Working Group on Obesity in China [13].

According to these criteria, all the participants were classified into four groups [19]: (1) MHO was designated for those with BMI ≥ 28 kg/m^2^ and none of the metabolically unhealthy parameters; (2) MUO represented those with BMI ≥ 28 kg/m^2^ and one or more metabolically unhealthy parameters; (3) participants with BMI < 28 kg/m^2^ and none of the metabolically unhealthy parameters were denoted as MHNO; (4) participants with BMI < 28 kg/m^2^ and one or more metabolically unhealthy parameters were denoted as MUNO.

Blood pressure (BP) was measured using electronic sphygmomanometers. BP was taken with the right upper arm kept at the level of the heart. After resting for 5 min, two measurements were taken at 1-min intervals, with the participants in a sitting position. If the difference between the two BP values was more than 10 mmHg, the measurement was recorded for the third time, and the final reading was the mean of the two closest measurements. Laboratory tests were conducted by laboratory physicians of Affiliated Hospital of Chengdu University per standard protocols. After overnight fasting of at least 8 h, venous blood was performed to measure FBG, TG, HDL-C, FBG, and other biochemical indicators.

#### Definition of CAP

A digital ultrasonic diagnostic system (EPIQ CX, Philips Ultrasound Inc., USA) was utilized to evaluate the presence/absence of CAP. The common carotid artery, the carotid artery bulb, and the internal carotid artery near and far wall segments were scanned bilaterally. The images were reviewed blindly by two physicians with more than five years of experience in vascular ultrasound imaging. According to the Mannheim criteria [20], CAP is defined as a focal region encroaching into the arterial lumen by at least 0.5 mm, > 50% of surrounding intima-media thickness values, or thickness ≥ 1.5 mm above the distance of the interface between the lumen-intima and the media-adventitia.

#### Covariates

Based on previously published studies [18, 21–23], indicators affecting the association between obese phenotype and CAP were considered covariates. In this respect, demographic characteristics (e.g., age), history of CVD (e.g., coronary atherosclerotic heart disease and myocardial infarction), lifestyle habits (e.g., smoking and alcohol drinking habits, and circadian rhythms), and some clinical biomarkers were collected by trained physicians and nurses to minimize bias. Circadian rhythm disorders were defined as working during the evening and overnight hours (6 P.M.–8 A.M) [24] and were self-reported by the participants. The working rhythms were also double-checked by the Social Security Department of Chengdu Railway Bureau. Trained investigators measured the body height and weight on standard methods. BMI was calculated as weight (in kilograms) divided by the square of height (in meters). Smokers were defined as those who smoked more than 1 cigarette/day for more than 1-year; other situations were considered nonsmokers. Alcohol drinkers were regarded as those drinking more than 1 time/week for over 6 months; other conditions were considered as nondrinkers. Some clinical biomarkers, such as serum uric acid (SUA), Total cholesterol (TC), and low-density lipoprotein cholesterol (LDL-C), were collected per standard protocols.

### Statistical analysis

According to the literature review data, the prevalence of CAP in the general Chinese population was 20.15% [25]. Assuming 80% power, a 2-sided α error of 0.05, and the allowable error was 3%; finally, a sample size of 687 was obtained. Considering a dropout rate of 20%, we decided on a minimum total sample size of 825. Thus, the sample size of our study has reached this standard.

Categorical variables were expressed as numbers and percentages, and the chi-square test was used to analyze differences in categorical variables. If the numerical values were not normally distributed, they were described as median (interquartile range) and analyzed by a rank-sum test. Multiple regression models were employed to estimate the associations between obese phenotypes and CAP risk after adjusting all the covariates, including age, TC, LDL-C, SUA, current smoking, drinking, history of CVD, and circadian rhythms. Subgroup analysis was performed by age, circadian rhythms, and history of drinking and smoking to investigate their modification effect. Odds ratios (ORs) and their 95% confidence intervals (CIs) to obtain the effect estimates.

Sensitivity analysis was conducted in this study. Two different criteria of obesity to classify the obese phenotypes, based on Asia Pacific criteria [26] and WHO criteria [27], were used to estimate the robustness of the results.

Two-sided *P* values were significant at less than 0.05. All statistical analyses were conducted in R Studio (Version 4.0.5).

## Results

### Baseline characteristics

A total of 8,645 subjects were enrolled in the study. There was a significant difference in the baseline characteristics among different obese phenotypes, including age, medical history of diabetes, hypertension, hyperlipidemia, and CVD (*P* < 0.001). The prevalence of CAP was higher in the metabolically unhealthy groups (MUNO 17.82% and MUO 18.67%) compared to the metabolically healthy groups (MHNO 5.36% and MHO 8.75%). (Table 1).

**Table 1:** Baseline characteristics of participants across different obese phenotypes (n = 8,645)

Variable	Median (p25, p75) or percentage (%)						P value
	**Total**	**MHNO**	**MHO**	**MUNO**		**MUO**	
	**N = 8,645**	** N = 2,257**	** N = 80**	** N = 5,360**		** N = 948**	
***Sociodemographics***							
**Age, year**	44	32	29	45		44	< 0.001
	(29.00, 48.00)	(25.00, 45.00)	(24.00, 43.00)	(38.00, 49.00)		(33.75, 48.00)	
**Age subgroup**							< 0.001
<45 years	4,716 (54.55)	1,640 (72.66)	61 (76.25)	2,489 (46.44)		526 (55.49)	
≥ 45 years	3,929 (45.45)	617 (27.34)	19 (23.75)	2,871 (53.56)		422 (44.51)	
***Medical history***							
Diabetes	404 (4.67)	0 (0.00)	0 (0.00)	345 (6.44)		59 (6.22)	< 0.001
Hypertension	1,071 (12.39)	0 (0.00)	0 (0.00)	801 (14.94)		270 (28.48)	< 0.001
Hyperlipidemia	316 (3.66)	0 (0.00)	0 (0.00)	231 (4.30)		85 (8.97)	< 0.001
CVD	76 (0.88)	13 (0.58)	0 (0.00)	52 (0.97)		11 (1.16)	< 0.001
***Lifestyle behaviors***							
Current smoker	5,404 (62.51)	1,257 (55.69)	49 (61.25)	3,474 (64.81)		624 (65.82)	< 0.001
Current drinker	1,420 (16.41)	194 (8.60)	10 (12.25)	1,039 (19.38)		177 (18.67)	< 0.001
Circadian rhythm disorders	4,929 (57.02)	1,334 (59.11)	46 (57.50)	2,975 (55.50)		574 (60.55)	< 0.001
***Clinical variables***							
BMI, kg/m^2^	24.04	22.03	29.37	24.13		29.46	< 0.001
	(21.99, 26.26)	(20.10, 23.95)	(28.58, 30.30)	(22.46, 25.79)		(28.68, 30.74)	
BMI < 28 kg/m^2^	7,617 (88.11)	2,257 (1.00)	0 (0.00)	5,360 (1.00)		0 (0.00)	< 0.001
28 ≤ BMI < 30 kg/m^2^	625 (7.23)	0 (0.00)	52 (65.00)	0 (0.00)		573 (60.44)	< 0.001
BMI ≥ 30 kg/m^2^	403 (4.66)	0 (0.00)	28 (35.00)	0 (0.00)		375 (39.56)	< 0.001
SBP, mmHg	124	114	118	128		132	< 0.001
	(114.00, 134.00)	(107.00, 120.00)	(112.75, 124.25)	(119.00, 137.00)		(124.00, 140.00)	
DBP, mmHg	81	73	75.5	85		88	< 0.001
	(74.00, 89.00)	(68.00, 78.00)	(70.75, 80.00)	(77.00, 91.00)		(81.00, 95.00)	
TC, mmol/L	4.65	4.27	4.43	4.77		4.87	< 0.001
	(4.08, 5.24)	(3.79, 4.80)	(3.95, 4.91)	(4.21, 5.38)		(4.33, 5.48)	
TG, mmol/L	1.59	1.06	1.31	1.9		2.3	< 0.001
	(1.10, 2.38)	(0.83, 1.30)	(1.15, 1.52)	(1.32, 2.65)		(1.67, 3.22)	
HDL-C, mmol/L	1.34	1.46	1.35	1.31		1.23	< 0.001
	(1.17, 1.54)	(1.31, 1.67)	(1.23, 1.50)	(1.13, 1.51)		(1.06, 1.38)	
LDL-C, mmol/L	2.89	2.53	2.78	2.98		3.15	< 0.001
	(2.41, 3.38)	(2.14, 2.97)	(2.48, 3.16)	(2.52, 3.48)		(2.74, 3.61)	
FBG, mmol/L	5.2	4.95	4.96	5.33		5.43	< 0.001
	(4.86, 5.63)	(4.68, 5.20)	(4.80, 5.22)	(4.95, 5.79)		(5.04, 6.01)	
SUA, mmol/L	387	367	438	389		434	< 0.001
	(337.00, 447.00)	(325.00, 419.00)	(381.00, 488.00)	(340.00, 447.00)		(374.00, 496.25)	
***Metabolic risk components***							
Elevated BP	4,021 (46.51)	0 (0.00)	0 (0.00)	3,334 (62.20)		687 (72.47)	< 0.001
Elevated FBG	2,301 (26.62)	0 (0.00)	0 (0.00)	1,894 (35.34)		407 (42.93)	< 0.001
Elevated TG	3,969 (45.91)	0 (0.00)	0 (0.00)	3,273 (61.06)		696 (73.42)	< 0.001
Reduced HDL-C	1,016 (11.75)	0 (0.00)	0 (0.00)	816 (15.22)		200 (21.10)	< 0.001
***Number of abnormal metabolisms***							< 0.001
1	2,702 (31.26)	0 (0.00)	0 (0.00)	2,451 (45.73)		251 (26.48)	
2	2,237 (25.88)	0 (0.00)	0 (0.00)	1,867 (34.83)		370 (39.03)	
3	1,170 (13.53)	0 (0.00)	0 (0.00)	899 (16.77)		271 (28.59)	
4	199 (2.30)	0 (0.00)	0 (0.00)	143 (2.67)		56 (5.91)	
***Outcome variables***							
CAP	1,260 (14.57)	121 (5.36)	7 (8.75)	955 (17.82)		177 (18.67)	< 0.001

BMI, body mass index; SBP, systolic blood pressure; DBP, diastolic blood pressure; TC, total cholesterol; TG, triglyceride; HDL-C, high-density lipoprotein cholesterol; LDL-C, low-density lipoprotein cholesterol; FBG, fasting blood glucose; SUA: serum uric acid; CAP: Carotid artery plaque; MHNO: metabolically healthy non-obesity; MHO: metabolically healthy obesity; MUNO: metabolically unhealthy non-obesity; MUO: metabolically unhealthy obesity

### Association between CAP and metabolic obese phenotypes

When compared with the MHNO group, the MUO group (2.20 [95%CI: 1.67, 2.89]) had a higher risk of CAP, followed by the MHO (2.18 [95%CI: 0.82, 5.10]) and MUNO (1.78 [95%CI: 1.44, 2.21]) groups after adjustment for age, TC, LDL-C, SUA, current smoking, current drinking, history of CVD, and circadian rhythms (Table 2). For the components of obese phenotypes, we observed that the BMI (1.04 [95%CI: 1.02, 1.06]), SBP (1.03 [95%CI: 1.02, 1.03]), DBP (1.04 [95%CI: 1.03, 1.04]) and FBG (1.14 [95%CI: 1.09, 1.18]) were significantly associated with CAP risk while no significant association was observed for TG and HDL-C (Table 3).

**Table 2:** Odds ratios and 95% confidence intervals for the risk of CAP across different obese phenotypes

		OR(95%CI)		
	**Crude model**	***P***-**value**	**Adjusted model** ^**a**^	***P***-**value**
**MHNO**	1.00 (ref.)		1.00 (ref.)	
**MHO**	1.69 (0.70, 3.51)	0.2	2.18 (0.82, 5.10)	0.09
**MUNO**	3.83 (3.16, 4.68)	< 0.001	1.78 (1.44, 2.21)	< 0.001
**MUO**	4.05 (3.17, 5.19)	< 0.001	2.20 (1.67, 2.89)	< 0.001

MHNO: metabolically healthy non-obesity; MHO: metabolically healthy obesity; MUNO: metabolically unhealthy non-obesity; MUO: metabolically unhealthy obesity. _a_ Adjustment for age, TC, LDL-C, SUA, current smoking, current drinking, history of CVD and circadian rhythms

**Table 3:** Association between components of obese phenotypes and CAP risk

		OR(95%CI)		
	**Crude model**	***P***-**value**	**Adjusted model** ^**a**^	***P***-**value**
**BMI**	1.07 (1.05, 1.09)	< 0.002	1.04 (1.02, 1.06)	< 0.001
**SBP**	1.05 (1.04, 1.05)	< 0.001	1.03 (1.02, 1.03)	< 0.001
**DBP**	1.06 (1.05, 1.07)	< 0.001	1.04 (1.03, 1.04)	< 0.001
**FBG**	1.29 (1.25, 1.34)	< 0.001	1.14 (1.09, 1.18)	< 0.001
**TG**	1.10 (1.07, 1.13)	< 0.001	1.02 (0.99, 1.06)	0.22
**HDL-C**	1.10 (0.91, 1.33)	0.34	0.84 (0.68, 1.05)	0.13

BMI, body mass index; SBP, systolic blood pressure; DBP, diastolic blood pressure; TG, triglyceride; HDL-C, high-density lipoprotein cholesterol; FBG, fasting blood glucose. ^a^ Adjustment for age, TC, LDL-C, SUA, current smoking, current drinking, history of CVD and circadian rhythms

### Subgroup analysis

**Fig. 1:**
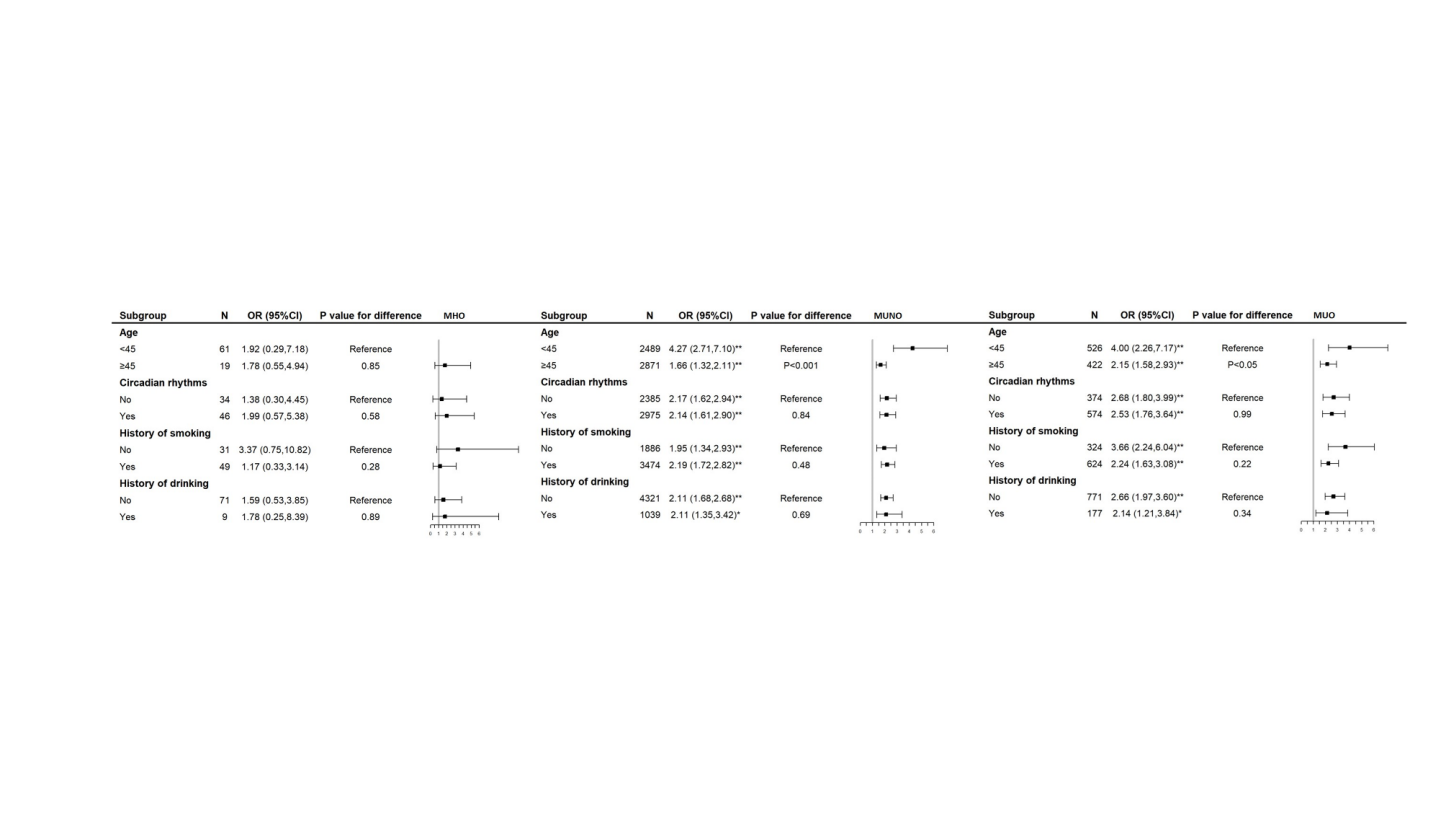
Subgroup analysis of the association of different obese phenotypes metabolic health and carotid artery plaque according to potential risk factors. MHNO: metabolically healthy non-obesity; MHO: metabolically healthy obesity; MUNO: metabolically unhealthy non-obesity; MUO: metabolically unhealthy obesity. The reference group was MHNO. Horizontal lines represent 95% confidence intervals. ^a^ Adjustment for age, TC, LDL-C, SUA, current smoking, current drinking, history of CVD, and circadian rhythms (except for the one used for stratification). ^*^P < 0.05, ^* *^P < 0.001

The risk of CAP was increased in both MUNO and MUO groups compared with the MHNO group (*P* < 0.01). Besides, those aged < 45 years with metabolically unhealthy conditions (MUNO and MUO groups) showed a higher risk of CAP, with OR (95%CI) of 4.27 (95%CI: 2.71, 7.10) and 4.00 (95%CI: 2.26, 7.17), respectively (*P* for difference < 0.05) **(**Fig. 1**)**.

### Sensitivity analysis

When using the Asia Pacific and WHO criteria for obesity, the results were robust **(**Table 4**)**. The metabolically unhealthy groups (MUNO and MUO) also had the highest risk of CAP compared with the MHNO group.

**Table 4:** Sensitivity analysis for the association between obese phenotypes and CAP risk based on different criteria of obesity

	Obesity with Asia Pacific criteria				Obesity with WHO criteria			
	**OR (95%CI)** ^**a**^				**OR (95%CI)** ^**a**^			
	**Crude model**	***P*** **-value**	**Adjusted model** ^**a**^	***P*** **-value**	**Crude model**	***P*** **-value**	**Adjusted model** ^**a**^	***P*** **-value**
**MHNO**	1.00 (ref.)		1.00 (ref.)		1.00 (ref.)		1.00 (ref.)	
**MHO**	1.61 (1.04, 2.42)	< 0.05	1.21 (0.76, 1.88)	0.42	2.11 (0.50, 6.13)	0.23	2.10 (0.49, 6.08)	0.23
**MUNO**	3.86 (3.10, 4.85)	< 0.001	1.72 (1.36, 2.20)	< 0.001	3.88 (3.21, 4.72)	< 0.001	3.85 (3.19, 4.68)	< 0.001
**MUO**	4.50 (3.62, 5.66)	< 0.001	2.02 (1.58, 2.60)	< 0.001	3.42 (2.45, 4.73)	< 0.001	3.39 (2.43, 4.69)	< 0.001

MHNO: metabolically healthy non-obesity; MHO: metabolically healthy obesity; MUNO: metabolically unhealthy non-obesity; MUO: metabolically unhealthy obesity. ^a^ Adjustment for age, TC, LDL-C, SUA, current smoking, current drinking, history of CVD, and circadian rhythms

## Discussion

In this cross-sectional study based on male railway drivers, we found that the risk of CAP was higher in metabolically unhealthy groups (MUO and MUNO) than in metabolically healthy groups (MHO and MHNO) and notably higher in metabolically unhealthy groups aged < 45 years. These findings could help to better understand the risk of CAP among male railway drivers and identify the groups of participants that need early health interventions.

Both metabolic abnormalities and obesity that can be expressed by obese phenotypes may exacerbate metabolic syndrome status and increase the risk of developing CAP [28]. In addition, we revealed that metabolically unhealthy groups were associated with a high risk of CAP, identical to previous studies on the general population. A retrospective cohort study with a sample size of 32,778 Chinese adults showed that different obese phenotypes were associated with the CAP risk [18]. However, the prevalence of CAP in male railway workers was significantly higher than that in previous studies (MHNO [5.36% vs. 1.1%], MHO [8.75% vs. 2.4%], MUNO [17.82% vs. 10.6%], MUO [18.67% vs. 6.3%], respectively) [29–31]. The differences may be explained by the high prevalence of unhealthy lifestyles and occupation-related characteristics among railway drivers (e.g. longer working hours, less physical activity, and circadian rhythm disorders). Previous studies have indicated that metabolically unhealthy patients had a high risk of CVD [14, 32, 33]. In contrast, the CVD risk among metabolically healthy individuals, such as MHO and MHNO groups, was controversial in previous studies [33, 34]. In this study, an association between metabolically healthy obese phenotypes and CAP risk among male railway drivers was found, and prior research involving a cohort of 3.5 million individuals reported a similar result [33]. Our study indicated that obese phenotypes could be used as a more precise classification of CAP risk in male railway workers.

The possible mechanism of the association varied among the obese phenotypes, and the risk of CAP can be explained by the following reasons. On the one hand, the common characteristics of obesity and metabolic abnormalities in lipid deposition in tissues lead to lipotoxicity, inflammation, and oxidative stress. All these factors can increase the risk of CAP [35, 36]. On the other hand, those with the metabolically healthy condition but obese phenotype were associated with lower levels of adiposity, which may explain their lower risk of carotid vascular endothelial injury than those with metabolic abnormalities. The available pieces of the literature showed a varied association between obese phenotypes and the risk of CVD [37, 38].

Our stratified analysis demonstrated that age modified the association between obese phenotypes and CAP, and those less than 45 years old had a high risk of CAP. The possible reason might be that drivers aged < 45 years had higher rates of poor lifestyles (e.g., for the prevalence of circadian rhythm disorders, 61.2% vs. 52.1%) and prevalence of obesity (12.4% vs. 11.22%) than those ≥ 45 years. Although circadian rhythms were not found as modifiers, circadian rhythms were a striking occupational characteristic among railway drivers. Circadian rhythms can affect atherosclerosis plaques through a neuro-immune axis that links sleep to hematopoiesis and atherosclerosis [29, 30]. While we did not observe a modification effect of circadian rhythms on the association between CAP risk and obese phenotypes, the MUO group with circadian rhythm disorders still had a 2.53-to-2.68-fold risk of developing CAP compared with the MHNO group. Further studies are needed to investigate the role of circadian rhythm disorders on the risk of CAP in railway drivers.

It should be noted that there were some limitations in the study. Firstly, caution should be taken in making causal interpretations between CAP risk and obese phenotypes since this study was a cross-sectional design. Further prospective studies are warranted to obtain the incidence of CAP in this population. Secondly, due to the occupational characteristics of railway workers, high pressure, and irregular lifestyles, practically all employees were male workers, which limited the generalization of our findings. For this reason, future multicenter studies are required to include female employees and extend these findings. Thirdly, although the results have adjusted for several important confounding factors, there still have many unselected or unmeasured factors, such as socioeconomic variables (e.g., income level, education level, exercise habit, etc.), some clinical biomarkers (e.g., creatinine, c-reactive protein, homocysteine, etc.), and personal history of diseases (e.g., chronic kidney disease). Therefore, further studies on this population are greatly needed to collect these confounding factors.

## Conclusion

This study is the first of its kind to investigate the association between obese phenotypes and CAP among male railway workers, and participants with MHO, MUNO, and MUO were associated with a high risk of CAP, especially in those with the metabolically unhealthy condition aged < 45 years.

## Data Availability

The datasets are available from the corresponding authors upon reasonable request.
