# Correction to: Association between obese phenotypes and risk of carotid artery plaque among chinese male railway drivers

**DOI:** 10.1186/s12889-023-15482-5

**Published:** 2023-06-30

**Authors:** Jia Pan, Zihang Wang, Chaohui Dong, Bo Yang, Lei Tang, Peng Jia, Shujuan Yang, Honglian Zeng

**Affiliations:** 1 Department of Health Management Center, Affiliated Hospital of Chengdu University, Chengdu, Sichuan China; 2 West China School of Public Health and West China Fourth Hospital, Sichuan University, Chengdu, Sichuan China; 3 School of Resource and Environmental Sciences, Wuhan University, Wuhan, China; 4 International Institute of Spatial Lifecourse Health (ISLE), Wuhan University, Wuhan, China

**Correction to**: ***BMC Public Health *22, 1859 (2022)**. 10.1186/s12889-022-14253-y

The original publication of this article contained 2 incorrect affiliations for Peng Jia. The updated affiliations are available via this correction article, the original article has been updated.

